# Effects of feeding an organic-certified milk replacer to preweaning Jersey dairy calves on growth performance, blood metabolites, and economic efficiency

**DOI:** 10.3168/jdsc.2025-0970

**Published:** 2026-03-19

**Authors:** O.R. Osuolale, C. LeCuyer, K.V. Almeida, L.H.P. Silva, R.C.R. Tinini, J.G. Dessbesell, A.F. Brito

**Affiliations:** 1 Department of Agriculture, Nutrition, and Food Systems, University of New Hampshire, Durham, NH 03824; 2 Universidade Estadual do Oeste do Paraná, Marechal Cândido Rondon, Paraná, Brazil, 85960-000

## Abstract

We aimed to evaluate the effects of feeding organic-certified raw whole milk (WM) versus organic-certified milk replacer (MR) on growth performance, skeletal development, and blood metabolites in preweaning Jersey calves, and the economic efficiency of these 2 liquid feeding regimens. Thirty organic-certified Jersey calves (18 females and 12 males) averaging (mean ± SD) at birth 24.8 ± 2.31 kg (heifers) and 28.7 ± 1.80 kg (bulls), were assigned to WM (n = 15) or MR (22.1% crude fat, 21.3% CP; n = 15) in a randomized complete block design. Calves were fed 4 L/d of WM or MR until weaning at 8 wk of age. Starter intake was greater in calves receiving MR (329 g/d) than WM (178 g/d), particularly from wk 4 to 8. Compared with WM calves, MR calves had lower final BW (49.3 vs. 54.7 kg) and ADG (411 vs. 483 g/d). Heart girth (80.8 vs. 82.8 cm), withers height (72.3 vs. 73.9 cm), hip height (75.1 vs. 76.6 cm), and body length (72.9 vs. 75.6 cm) were all lower in MR than WM calves at wk 8 of age, with treatment by week interactions observed for heart girth and withers height. In addition, the plasma concentrations of glucose (98.8 vs. 106 mg/dL) and urea N (8.34 vs. 9.66 mg/dL) were lower with feeding MR than WM, with a treatment by week interaction seen for plasma urea N. However, the plasma concentrations of BHB and ketones did not differ between treatments. The economic analysis showed that feeding MR was less cost-effective than feeding WM due to increased total feed costs per calf (median = $228.89 vs. $157.31) and per kilogram of BW gain (median = $9.94 vs. $5.82). In conclusion, feeding MR to preweaning organic-certified Jersey calves reduced ADG, skeletal development, and profitability compared with WM.

## Highlights

• Starter intake was greater in calves fed MR than in those fed WM.
• Calves fed MR gained less weight compared with those fed WM.
• Skeletal growth was slower in calves fed MR versus WM.
• Feeding MR was less profitable relative to WM.

## Graphical Abstract

This study evaluated the effects of feeding an organic-certified raw whole milk (WM) or an organic-certified milk replacer (MR) on growth, skeletal development, and blood metabolites in preweaning Jersey calves. We also evaluated the economic efficiency of feeding WM versus MR to dairy calves. Calves fed MR had lower average daily gain and skeletal development compared with those fed WM. The economic analysis showed that feeding MR was less cost effective than WM because of increased cost per calf and per kilogram of body weight gain. In brief, our findings revealed that MR was not an effective feeding strategy for organic-certified dairy calves, underscoring the need for better formulations of MR to be used on organic dairy farms.

**Figure:**
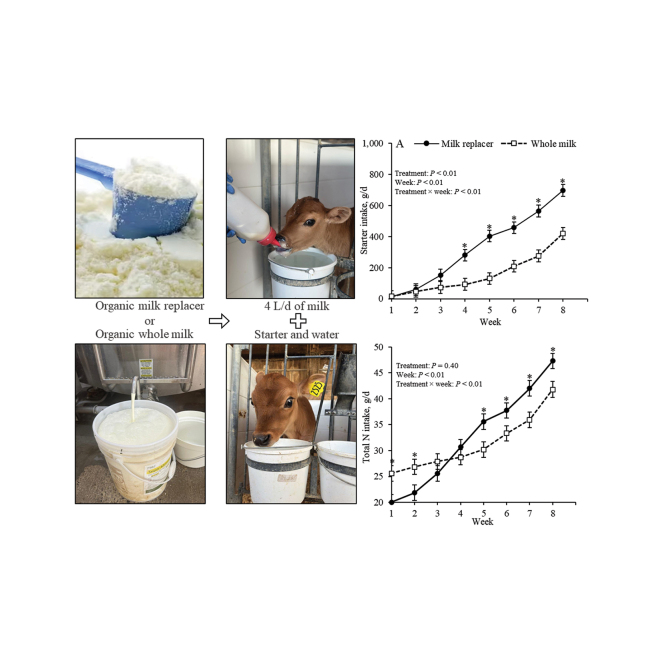

The organic dairy sector has grown substantially in the United States, due primarily to increased consumers' demand, with farm-level sales rising 88% from 2008 to 2021 (Gillespie et al., 2024). Organic-certified dairy systems are regulated by a set of federally-mandated rules that prohibit the use of antibiotics for treating animals and chemical or synthetic fertilizers for boosting crops production (USDA-AMS, 2000). In addition, ruminants >6 mo of age must have year-round access to the outdoors and consume 30% of their total DMI from grazed herbage for a minimum of 120 d during the year (USDA-AMS, 2010). Therefore, organic dairy producers in the United States must have functional pasture-based systems in place to raise heifers after weaning to comply with federal regulations regarding herbage DMI, among other mandates.

High-quality liquid feeding during the preweaning period is essential for ensuring health, growth, and long-term productivity of dairy calves (Soberon et al., 2012). Specific to Jerseys, ADG has been reported to range from 560 to 584 g/d in conventional calves fed milk replacer (**MR**; 28.0% crude fat, 28.2% CP) versus pasteurized saleable whole milk (**WM**; Bowen Yoho et al., 2013), and from 370 to 520 g/d in organic-certified calves fed raw WM (Stahl et al., 2026). Whole milk is usually regarded as the “gold standard” for calf nutrition due to the high biological value and bioavailability of its essential nutrients that may be difficult to replicate with substitutes like MR (Lee et al., 2009). Nevertheless, MR offer practical advantages, including reduced use of saleable milk and improved biosecurity by minimizing disease transmission (Cowles et al., 2006). Organic dairy producers have traditionally relied on WM to raise calves due to the limited availability of organic-certified MR on the market (Stiglbauer et al., 2013). However, we are aware of only 1 published study (Sharpe and Heins, 2021) that has compared raw WM versus MR (22% crude fat, 20% CP) as liquid feeding strategies to organic-certified dairy calves in an automated milk feeding system. In that study (Sharpe and Heins, 2021), they showed that group-housed Holstein and crossbred dairy calves fed MR or WM had similar ADG, weaning BW, and skeletal development despite greater average cost per day ($6.06 vs. $4.47) and average cost per kilogram of BW gain ($8.83 vs. $6.39) with feeding MR versus WM. In contrast, Godden et al. (2005) showed lower ADG in conventional dairy calves fed MR (20% crude fat, 20% CP) compared with pasteurized nonsaleable WM, indicating that further research is needed, particularly in organic dairy systems because of limited published data.

We hypothesized that feeding organic-certified MR would result in lower ADG and skeletal growth than organic-certified WM because of MR being less energy and nutrient dense than raw WM. Our objective was to compare the effects of feeding WM versus MR on growth performance, skeletal development, and blood metabolites in preweaning Jersey calves raised in a manual liquid feeding regime. A second objective was to evaluate the economic efficiency of feeding WM versus MR to preweaning Jersey calves raised in an organic dairy system.

All experimental procedures involving animal care and management were reviewed and approved by the University of New Hampshire Institutional Animal Care and Use Committee (protocol no. 171002). This study was conducted at the University of New Hampshire Burley-Demeritt Organic Dairy Research Facility (Lee, NH).

Thirty organic-certified dairy calves (n = 18 females and 12 males) born between November 2017 and April 2018 and averaging (mean ± SD) at birth 24.8 ± 2.31 kg (heifers) and 28.7 ± 1.80 kg (bulls) were used in the study. After birth, newborn calves were separated from their dams, vaccinated against *Escherichia coli* (Bar-Guard-99; Boehringer Ingelheim) and bovine rota- and coronavirus (Calf-Guard; Zoetis), and navel-dipped with a 7% (vol/vol) iodine tincture. Calves were then housed in individual pens (1.50 × 0.97 m) inside of a 3-sided, naturally-ventilated calf barn. The stalls were cleaned twice daily by removing feces and visibly wet or soiled bedding, with fresh kiln-dried sawdust added as needed. Insulated jackets and heat lamps were provided during the winter months for supplemental warmth. Each calf received 4 L of high-quality maternal colostrum (>50 g/L IgG), measured with a colostrometer (Biogenics), and delivered via nipple bottles within the first 12 h of life. When the entire volume of colostrum was not consumed in the 12-h time allotted, the remaining amount was administered through an esophageal tube.

On d 2 of life, calves were blocked in pairs by birth date (n = 15 blocks) and, within block, assigned randomly to raw WM (n = 15; 9 heifers and 6 bulls) or an organic-certified MR (Organi-Calf Instant Milk; Milk Specialties Global; n = 15; 9 heifers and 6 bulls). Calves in both treatments were fed 4 L/d of WM or MR split equally into 2 feedings at 0630 and 1800 h. The allotted portions of WM or MR were consumed entirely by all calves. Whole milk was obtained from the bulk tank and contained (mean ± SD), on average, 14.0% ± 0.66% TS, 4.74% ± 0.08% lactose, 4.73% ± 0.63% fat, and 3.70% ± 0.26% true protein, yielding (DM basis) 33.7% fat and 27.9% CP. The MR fed was a milk protein-whey-palm oil formula (22% crude fat, 21% CP), which was prepared following the manufacturer's guidelines by diluting 286 g of the dry product in 1.89 L of warm tap water aiming for a TS concentration of 14.7%. Calves had free choice access to a pelleted starter grain (Organic Calf Starter 20% CP Pellet; Morrison's Custom Feeds Inc., Barnet, VT) containing (% as fed): 20.8% ground corn, 20% wheat middlings, 18.2% soybean meal, 12.4% barley, 10% canola meal, 9.3% roasted soybean, 6% sugarcane molasses, 0.9% ground limestone, 0.70% *Ascophyllum nodosum* meal (kelp meal), 0.50% salt, 0.40% mineral-vitamins premix, 0.40% AB-20 (aflatoxin binder; PrinceAgri Products Inc.), 0.40% mannan oligosaccharides, and 0.10% vitamin E. Starter offered and refused were recorded at each feeding using a digital hanging scale to calculate starter intake after drying the samples at 55°C for 48 h to determine DM. Water offered and refused were weighed concurrently with starter to quantify individual water intake.

Body weight was recorded at birth and weekly thereafter, with total BW gain calculated by subtracting weaning BW from birth BW. Average daily weight gain was determined by subtracting weaning BW from birth BW and dividing by 56 d. Skeletal measurements were recorded at birth and weekly subsequently, with withers height, hip width, and hip height measured using a sliding-scale height stick with a bubble level (Nasco Education). Body length and heart girth were measured with a weight tape (Nasco Education). Fecal score (1 = normal consistency/firm feces, 2 = semiformed or pasty and loose feces, and 3 = watery feces) was recorded daily throughout the experiment to monitor scours using a 1 to 3 score system adapted from McGuirk (2018). One heifer assigned to the MR treatment experienced severe diarrhea and was treated with injections of 3 mL vitamin C, 0.5 mL vitamin E/Se (BO-SE; Merck Animal Health), and 7 mL of an antibacterial blend of botanicals (GetWell; Cow Master LLC) for 3 d. Data collected during the week in which this heifer was clinically ill were excluded from the statistical analyses, with remaining data kept in the datasets for running statistics.

Bulk tank milk samples were collected weekly throughout the study, preserved with 2-bromonitropropane-1,3 diol (Broad Spectrum Microtabs II; Advanced Instruments Inc.), and shipped to Dairy One DHIA Laboratory (Ithaca, NY) for analyses of fat, true protein, lactose, and TS via Fourier transform infrared spectroscopy using a MilkoScan FT+ (Foss Inc.). Milk replacer samples (n = 6) were collected every time a new bag was opened and shipped to Dairy One Forage Testing Laboratory (Ithaca, NY) for nutrient analyses using AOAC International (2016) methods, except otherwise noted, as follows: DM (method 930.15), CP (method 990.03), soluble CP (Krishnamoorthy et al., 1982), NDF (Ankom Technology method 15 in an Ankom^200^ Fiber Analyzer; solutions based on Van Soest et al., 1991), ADF (Ankom Technology method 14 in an Ankom^200^ Fiber Analyzer; solutions based on method 973.18), crude fat (method 2003.05), and ash (method 942.05). Minerals (Ca, P, Mg, K, Na, Fe, Zn, Cu, Mn, Mo, and S; only Ca and P shown) were quantified with an iCAP 6300 Intrepid Inductively Coupled Plasma Radial Spectrometer (Thermo Fisher Scientific Inc.) after microwave digestion. Samples of starter pellets (n = 10) were collected during the study and shipped to Dairy One Forage Testing Laboratory and analyzed for the same nutrients (except soluble CP, crude fat, and S) and by the same methods used for MR. The actual nutrient composition of the MR used in the present study averaged (DM basis; mean ± SD) 21.3% ± 0.39% CP, 76.8% ± 3.31% soluble CP (% of CP), 22.1% ± 0.29% crude fat, 6.57% ± 0.11% ash, 1.29% ± 0.03% Ca, 0.92% ± 0.04% P, and 0.66 ± 0.004 Mcal/kg NEG, with that of starter averaging (DM basis) 24.9 ± 1.07% CP, 14.4 ± 0.98% NDF, 6.90 ± 0.70% ADF, 6.75 ± 0.48% ash, 0.86 ± 0.21% Ca, 0.52 ± 0.06% P, and 0.41 ± 0.009 Mcal/kg NEG.

Blood samples were collected approximately 4 h after colostrum feeding, and weekly at 1300 h for the duration of the experiment via jugular venipuncture using 10-mL vacutainer tubes (Becton Dickinson) with EDTA and without preservatives and analyzed for ketones, glucose, plasma urea N (**PUN**), and BHB. A single drop of whole blood from the vacutainer tubes without preservatives was transferred using a disposable pipette onto the test strip of a hand-held electronic ketones monitoring device (Nova Max Plus; Nova Biomedical) to determine total ketones concentration. The EDTA tubes were transported to the laboratory refrigerated on ice, with blood samples centrifuged (model no. 5810; Eppendorf North America) at 2,155 × *g* at 4°C for 20 min. Plasma was harvested and frozen at −20°C until analyzed for glucose (kit no. 700190; Cayman Chemical Inc.), PUN (kit no. 0580; Stanbio Laboratory Inc.), and BHB (kit no. 700190; Cayman Chemical Inc.) using colorimetric assays on a UV/visible spectrophotometer (BioTek Instruments Inc.) and read at wavelengths of 520, 520, and 455 nm, respectively.

Milk costs were determined by expenses associated with feeding WM and MR from d 2 to d 56 of life. Whole milk was set at $0.65/kg based on the average organic milk price received in the Northeastern United States during the course of the study, with MR set at $6.61/kg in line with the original price paid at purchase. Starter costs were calculated based on starter intake with a current market price of $1.02/kg. Milk cost per calf was calculated by multiplying the price per kilogram of WM or MR by their amounts consumed during the study, with values converted to a DM basis (WM = 12.5% DM; MR = 94.6% DM) to allow direct comparisons between treatments. Total feed cost per calf was obtained by the sum of WM or MR and starter grain costs, and total feed cost per kilogram of BW gain was calculated by dividing the total feed cost per calf by the total BW gained. A sensitivity analysis was performed to account for cost variability using a ±10% price range.

Data were analyzed according to a randomized complete block design with repeated measures over time using the MIXED procedure of SAS (version 9.4; SAS Institute Inc.). Week was used as the repeated term for intake, skeletal measurements, and blood metabolites. Calf sex, as well as birth BW and skeletal measurements were tested as covariates and excluded from the model if *P* > 0.25. The following statistical model was used to analyze the data:

Y_ijkl_ = µ + B_i_ + TRT_j_ + W_k_ + βC_ijkl_ + TRT_j_ × W_k_ + e_ijkl_,

where Y_ijkl_ is the dependent variable, µ is the overall mean, B_i_ is the random effect of the ith block (i = 1, . . ., 15), TRT_j_ is the fixed effect of the jth treatment (j = WM vs. MR), W_k_ is the fixed effect of the kth week (k = 1, . . ., 8), β is the regression coefficient of the covariate C, C_ijkl_ is the covariate variable for the lth calf within the ith block and jth treatment in the kth week, TRT_j_ × W_k_ is the interaction between the jth treatment and the kth week, and e_ijkl_ is the residual error term. The SAS command REPEATED was used to model distinct residual variances, and among the covariance structures tested (i.e., spatial power, compound symmetry, autoregressive [1], and heterogeneous autoregressive [1]), the one with the lowest Bayesian information criterion was retained in the final model. The SAS PDIFF option was used to separate treatment means when a treatment by week interaction was present (*P* ≤ 0.05).

A treatment by week interaction was observed for starter DMI (*P* < 0.001; Table 1), with calves fed MR consuming more starter than those fed WM from wk 4 to wk 8 of age (Figure 1A). Increased starter DMI in calves receiving MR is likely explained by the lower energy concentration and nutrient bioavailability of MR compared with WM (digestible energy = 4.92 vs. 5.49 Mcal/kg; NASEM, 2021), resulting in MR calves ingesting more solid feed to compensate for the energy deficit. In agreement with our results, Wang et al. (2022) reported greater starter DMI (230 vs. 160 g/d) during the preweaning phase in Holstein calves fed MR versus WM powder, with diets formulated to be isocaloric and isonitrogenous and milk offered at a rate of 8 L/d. In contrast, Wilms et al. (2022) observed no difference in starter DMI (mean = 363 g/d) when comparing WM powder and 3 MR containing different levels of crude fat, lactose, and CP fed to preweaning Holstein calves, with milk intake varying from 8.29 L/d (WM powder) to 9.65 L/d (high-lactose MR diet). We also detected treatment by week interactions (*P* < 0.01; Table 1) for total DMI and total ME intake, with total DMI greater (*P* < 0.001) in MR than WM calves from wk 4 to wk 8 and total ME intake (*P* ≤ 0.02) from wk 5 to wk 8 (data not shown). Furthermore, water intake was 49.4% lower (*P* < 0.01) in MR versus WM calves (Table 1). Similarly, Wilms et al. (2022) showed that preweaning calves fed MR drank, on average, 47.6% more water than those fed WM powder. In the present experiment and in that of Wilms et al. (2022), PUN and serum urea concentrations were greater (discussed later in this article) in calves fed WM (liquid or powder) versus those receiving MR. It is conceivable that increased circulating urea in WM-fed calves across these 2 studies may have elevated urinary output to excrete excess N, resulting in more water consumption to compensate for water losses via urine.

**Table 1:** Intake, growth performance, skeletal measurements, and blood metabolites in preweaning Jersey calves fed organic-certified raw whole milk (WM) versus an organic-certified milk protein-whey-palm oil-based milk replacer (MR) from birth to 8 wk of age

Item	Treatment (TRT)		SEM	*P-*value		
	WM (n = 15)	MR (n = 15)		TRT	Week	TRT × week
Birth BW, kg	26.5	26.2	0.56	0.66	—	—
Intake						
Water, g/d	1,021	517	129	<0.01	<0.001	0.23
Liquid feed DMI, g/d	561	571	—	—	—	—
Starter DMI, g/d	158	329	28.5	<0.001	<0.001	<0.001
Total DMI, g/d	718	900	28.5	<0.001	<0.001	<0.001
Total N intake, g/d	31.3	32.6	1.14	0.40	<0.001	<0.001
Total ME intake,^1^ g/d	3.48	3.79	0.13	0.09	<0.001	<0.01
Growth performance						
Final BW, kg	54.7	49.3	1.32	0.01	—	—
ADG, g/d	483	411	21.2	0.03	—	—
ADG/total DMI, g/g	0.67	0.45	0.01	<0.001	—	—
Skeletal measurement						
Heart girth, cm	82.8	80.8	0.43	<0.001	<0.001	<0.01
Withers height, cm	73.9	72.3	0.27	<0.001	<0.001	<0.01
Hip height, cm	76.6	75.1	0.26	<0.001	<0.001	0.52
Hip width, cm	18.3	18.0	0.22	0.18	<0.001	0.91
Body length, cm	75.6	72.9	0.52	<0.001	<0.001	0.17
Blood metabolites						
Glucose, mg/dL	106	98.8	2.24	0.02	<0.001	0.14
PUN, mg/dL	9.66	8.34	0.37	<0.001	0.05	0.01
BHB, m*M*	0.126	0.117	0.006	0.25	<0.001	0.46
Ketones, m*M*	0.199	0.183	0.016	0.44	0.02	0.55
Fecal score,^2^ points	1.06	1.54	0.05	<0.001	<0.001	<0.001

1 Estimated using NASEM (2021).

2 Fecal score: 1 = normal consistency/firm feces; 2 = semiformed or pasty and loose feces; and 3 = watery feces.

**Figure 1:**
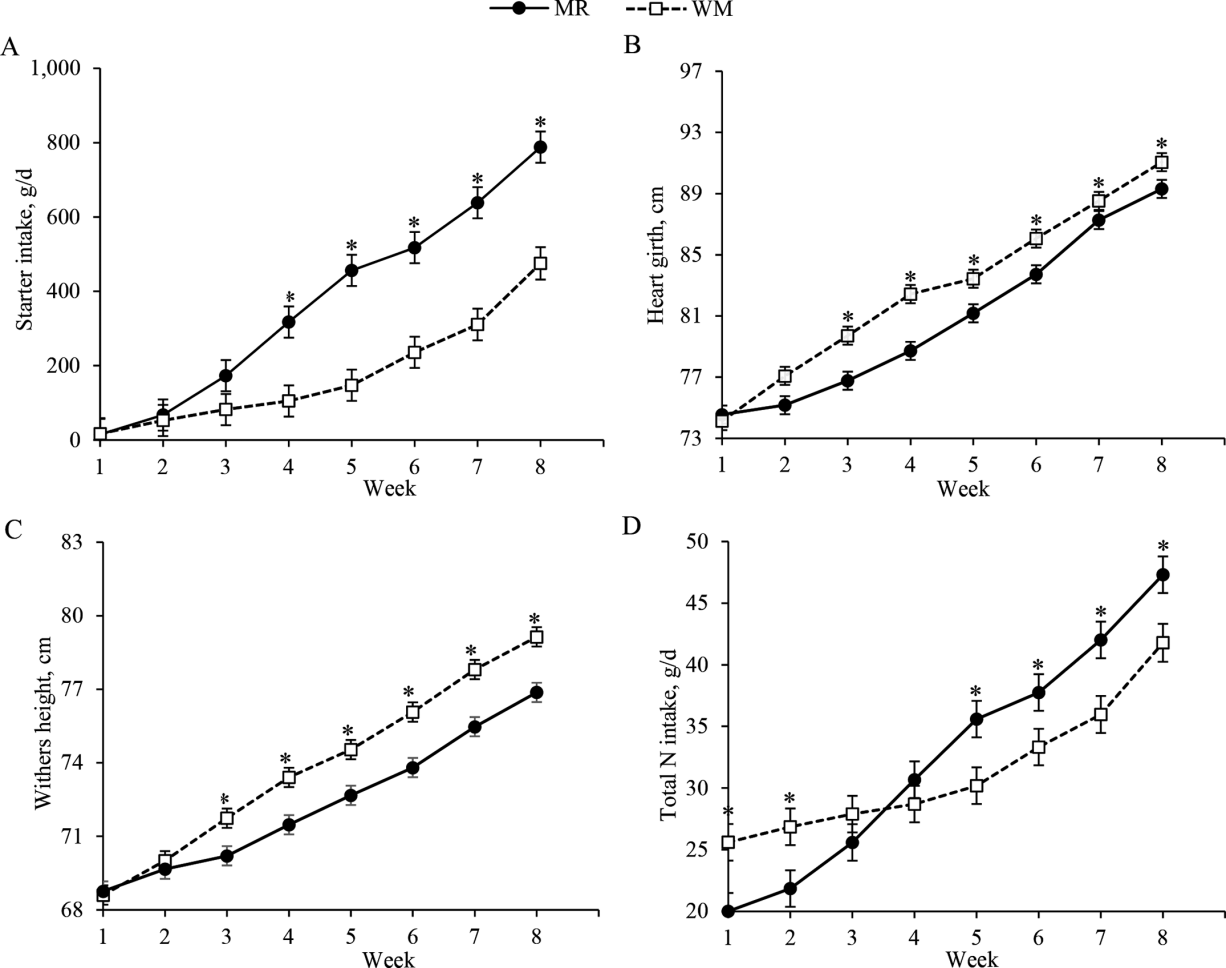
Starter DMI (A), heart girth (B), withers height (C), and total N intake (D) in preweaning Jersey calves fed organic-certified raw whole milk (WM; n = 15) versus an organic-certified milk protein-whey-palm oil-based milk replacer (MR) from birth to 8 wk of age. *Indicates difference between treatments within week (*P* ≤ 0.05). Error bars represent SEM.

Calves fed MR were 9.87% lighter at wk 8 of age (*P* = 0.01) than those fed WM despite MR calves consuming more starter DM than their WM counterparts (Table 1). In addition, both ADG (−14.9%) and feed efficiency (ADG/total DMI; −32.8%) were lower (*P* ≤ 0.03) with feeding MR versus WM. Altogether, these results suggest that less energy and AA were partitioned toward BW gain in MR than WM calves, likely explaining their lowest weaning BW, ADG, and feed efficiency despite greater ME intake (wk 5–8) in MR versus WM diet. Wang et al. (2022) also observed that the ADG (830 vs. 920 g/d) and feed efficiency (0.39 vs. 0.43 g/g) were lower in preweaning calves receiving MR versus WM powder. In contrast, Wilms et al. (2022) reported that the ADG was lowest in calves fed WM powder (889 g/d) and fat-based MR (955 g/d), intermediate in those receiving MR enriched with protein (973 g/d), and greatest in the lactose-based MR calves (1,057 g/d). They also showed that feed efficiency (calculated as ME intake/ADG) was greatest (30.7 MJ/kg) and lowest (26.5 MJ/kg) in WM and lactose-based MR calves, respectively (Wilms et al., 2022). Sharpe and Heins (2021) saw no differences in ADG (mean = 715 g/d) and feed efficiency (mean = 0. 81 g/g) in preweaning, organic-certified Holstein and crossbred calves offered 8 L/d of WM or the same MR product used herein. Discrepancies seen for the ADG results among experiments may be because feeding a larger (≥8 L/d; Sharpe and Heins, 2021; Wilms et al., 2022) versus a smaller (4 L/d; present study) volume of liquid feed likely offset potential differences in nutrient utilization between WM and MR. However, Moallem et al. (2010) observed lower ADG (−9.17%) and feed efficiency (−19.2%) in Holstein calves fed MR versus WM despite milk intake of 9.78 and 8.97 L/d, respectively. Alternatively, MR supplementation with fat, lactose, and protein as evaluated in the Wilms et al. (2022) study possibly counteracted the differences in nutrient profile of MR versus WM powder.

We observed treatment by week interactions (*P* < 0.01; Table 1) for heart girth (Figure 1B) and withers height (Figure 1C), with these 2 variables showing lower values in calves fed MR versus WM from wk 3 to wk 8. In addition, calves fed MR had lower (*P* < 0.001) hip height and shorter (*P* < 0.001) body length than those offered WM, suggesting that MR calves were less efficient to convert ME into skeletal growth. Contrarily, hip width was not affected (*P* = 0.18) by treatments. Wang et al. (2022) reported lower withers height (94.6 vs. 97.2 cm) and heart girth (107.1 vs. 109.9 cm) with feeding MR versus WM powder in calves at 70 d of age, thus in agreement with the present results. However, Sharpe and Heins (2021) observed no differences in hip height (mean = 91.5 cm) and heart girth (mean = 101.0 cm) at weaning in calves fed WM or MR. In contrast to Sharpe and Heins (2021), Wang et al. (2022), and the present study, Wilms et al. (2022) showed that feeding WM powder resulted in lower withers height (mean = −1.61%), hip height (mean = −1.60%), and heart girth (mean = −2.22%) at preweaning and weaning compared with the average of 3 MR. As discussed earlier, supplementation with fat, lactose, and protein improved the nutritional quality of the MR fed in Wilms et al. (2022) study, highlighting the potential shortage of digestible energy and AA in nonsupplemented MR relative to WM.

The plasma concentration of glucose was 6.79% lower (*P* = 0.02) in calves fed MR than WM (Table 1), possibly reflecting the reduced concentration of lactose in MR compared with WM. In support, Welboren et al. (2021) showed that the baseline glucose concentration was greater in Holstein calves receiving a high-lactose MR (46.1% lactose and 18% fat) than a high-fat MR (39.9% lactose and 24.6% fat) at d 6 of life. However, a 17.4% increase in lactose concentration in a lactose-based MR compared with WM powder did not change the blood concentration of glucose in preweaning calves (Wilms et al., 2022), suggesting that other factor(s) beyond lactose supply may have been involved. A treatment by week interaction was detected for PUN concentration (*P* = 0.01; Table 1), with MR calves showing lower values in wk 1, 2, 3, and 5 than WM calves (data not shown). Similarly, a treatment by week interaction (*P* < 0.001) was observed for total N intake (Table 1). Whereas MR calves had lower (*P* ≤ 0.02) N intake than WM calves during wk 1 and 2, they consumed more N (*P* ≤ 0.03) from wk 5 to 8 (Figure 1D). Although lowered N intake is in line with reduced PUN concentration during wk 1 and 2 in the MR diet, PUN either decreased (wk 5) or did not change (wk 6–8) despite greater N intake with feeding MR versus WM. These results suggest that compared with the WM diet, protein from MR and the additional starter grain consumed in the MR diet was possibly less degraded in the gastrointestinal tract leading to reduced PUN levels. Wilms et al. (2022) showed that the serum concentration of urea was, on average, 27.2% lower in calves fed fat- and lactose-based MR than WM powder. According to Wilms et al. (2022), the increased serum urea concentration with feeding WM powder could be related to WM-protein denaturation reducing its biological value or increased WM-protein breakdown. Treatments did not affect (*P* ≥ 0.25) the plasma concentrations of BHB and ketones (Table 1), implying similar ruminal development between diets even though starter DMI was greater in MR than WM calves.

The median milk, starter, and total feed costs were 41.2%, 109%, and 45.5% greater, respectively, with feeding MR versus WM to Jersey calves (Table 2). In addition, the median cost per kilogram of BW gain averaged $9.94 and $5.82 for MR versus WM calves, indicating that feeding MR was 41.4% less cost-effective to promote gain than feeding WM, in line with the economic analysis done by Sharpe and Heins (2021), who compared WM and the same MR fed in our study. Our results further revealed that even when MR costs dropped from $228.75 to $187.16/calf and WM costs increased from $132.56 to $162.02/calf, WM remained the more cost-effective option ($6.40 vs. $8.95/kg of BW gain).

**Table 2:** Economic analysis of organic-certified raw whole milk (WM) versus an organic-certified milk protein-whey-palm oil-based milk replacer (MR) fed to preweaning Jersey calves from birth to 8 wk of age

Item	WM (n = 15)			MR (n = 15)		
	Lower^1^	Median	Upper^1^	Lower^1^	Median	Upper^1^
Liquid feed costs, $/calf	132.56	147.29	162.02	187.16	207.95	228.75
Starter costs, $/calf	9.02	10.02	11.02	18.84	20.94	23.03
Total feed costs, $/calf	141.58	157.31	173.04	206.00	228.89	251.78
BW gain, kg	27.1	27.1	27.1	23.0	23.0	23.0
Cost per BW gain, $/kg	5.23	5.82	6.40	8.95	9.94	10.94

1 Lower and upper values represent cost variability using a ±10% sensitivity range.

In conclusion, Jersey calves fed 4 L/d of an organic-certified MR in a manual liquid feeding system had lower ADG, feed efficiency, and skeletal growth than those fed organic WM. Although MR-fed calves consumed more starter DM, the plasma concentration of BHB did not differ between treatments, suggesting similar ruminal development until 8 wk of age. Feeding MR was also more expensive than WM, which can negatively affect the profitability of organic dairy farms.
